# Erratum to: An egalitarian society? Widening inequalities in premature mortality from non-communicable diseases in Australia, 2006-16

**DOI:** 10.1093/ije/dyab040

**Published:** 2021-03-19

**Authors:** Tim Adair, Alan D Lopez

First published online: 8 December 2020, *Int J Epidemiol* 2020; doi: https://doi.org/10.1093/ije/dyaa226

In the text of the results, several negative values in confidence intervals were published as positive values, and a hyphen was missing from a range of years indicating 2011 to 2016.

These errors have now been corrected.

